# Facile method to synthesize magnetic iron oxides/TiO_2 _hybrid nanoparticles and their photodegradation application of methylene blue

**DOI:** 10.1186/1556-276X-6-533

**Published:** 2011-09-30

**Authors:** Wei Wu, Xiangheng Xiao, Shaofeng Zhang, Feng Ren, Changzhong Jiang

**Affiliations:** 1 Key Laboratory of Artificial Micro- and Nano-structures of Ministry of Education, Wuhan University, Wuhan 430072, People's Republic of China; 2 Center for Electron Microscopy and School of Physics and Technology, Wuhan University, Wuhan 430072, People's Republic of China; 3 School of Printing and Packaging, Wuhan University, Wuhan 430079, People's Republic of China China

**Keywords:** magnetic iron oxide nanoparticles, TiO_2_, hybrid structure, photocatalyst, methylene blue

## Abstract

Many methods have been reported to improving the photocatalytic efficiency of organic pollutant and their reliable applications. In this work, we propose a facile pathway to prepare three different types of magnetic iron oxides/TiO_2 _hybrid nanoparticles (NPs) by seed-mediated method. The hybrid NPs are composed of spindle, hollow, and ultrafine iron oxide NPs as seeds and 3-aminopropyltriethyloxysilane as linker between the magnetic cores and TiO_2 _layers, respectively. The composite structure and the presence of the iron oxide and titania phase have been confirmed by transmission electron microscopy, X-ray diffraction, and X-ray photoelectron spectra. The hybrid NPs show good magnetic response, which can get together under an external applied magnetic field and hence they should become promising magnetic recovery catalysts (MRCs). Photocatalytic ability examination of the magnetic hybrid NPs was carried out in methylene blue (MB) solutions illuminated under Hg light in a photochemical reactor. About 50% to 60% of MB was decomposed in 90 min in the presence of magnetic hybrid NPs. The synthesized magnetic hybrid NPs display high photocatalytic efficiency and will find recoverable potential applications in cleaning polluted water with the help of magnetic separation.

## Introduction

Extended and oriented nanostructures are desirable for many applications, but facile fabrication of complex nanostructures with controlled crystalline morphology, orientation, and surface architectures remains a significant challenge [1]. Among their various nanostructured materials, magnetic NPs-based hybrid nanomaterials have attracted growing interests due to their unique magnetic properties. These functional composite NPs have been widely used in various fields, such as magnetic fluids, data storage, catalysis, target drug delivery, magnetic resonance imaging contrast agents, hyperthermia, magnetic separation of biomolecules, biosensor, and especially the isolation and recycling of expensive catalysts [2-12]. To this end, magnetic iron oxide NPs became the strong candidates, and the application of small iron oxide NPs has been practiced for nearly semicentury owing to its simple preparation methods and low cost approaches [13].

Currently, semiconductor NPs have been extensively used as photocatalyst. TiO_2 _NPs have been used as aphotocatalytic purification of polluted air or wastewater, will become a promising environmental remediation technology because of their high surface area, low cost, nontoxicity, high chemical stability, and excellent degradation for organic pollutants [14-17]. Moreover, TiO_2 _also bears tremendous hope in helping to ease the energy crisis through effective utilization of solar energy based on photovoltaic and water-splitting devices [18-21]. As comparing with heterogeneous catalysts, many homogenerous catalytic systems have not been commericalized because of one major disadvantage: the difficulty of separation the reaction product from the catalyst and from any reaction solvent for a long and sustained environment protection [22]. In addition, there are two bottleneck drawbacks associated with TiO_2 _photocatalysis currently, namely, high charge recombination rate inherently and low efficiency for utilizing solar light, which would greatly hinder the commercialization of this technology [23]. Currently, the common methods are metals/non-metals-doping or its oxides-doping to increasing the utilization of visible light and enhancing the separation situation of charge carriers [24-27]. More importantly, the abuse and overuse of photocatalyst will also pollute the enviroment.

**Figure 1:**
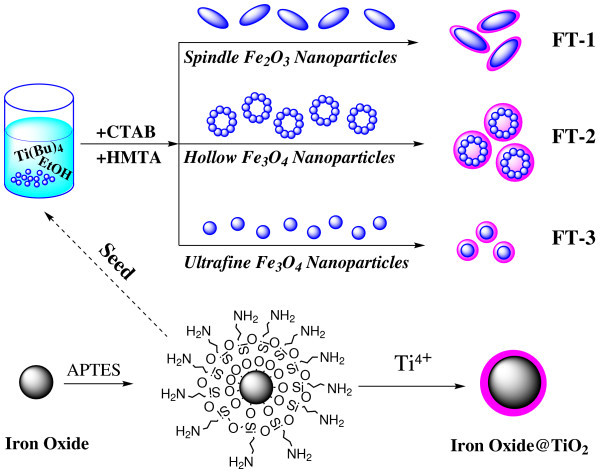
**Illustration of the synthetic chemistry and process of magnetic iron oxide/TiO_2 _hybrid NPs preparation**.

In this point, magnetic separation provides a convenient method to removing pollutants and recycling magnetized species by applying an appropriate external magnetic field. Therefore, immobilization of TiO_2 _on magnetic iron oxide NPs has been investigated intensely due to its magnetic separation properties [28-32]. Indeed, the study of core-shell magnetic NPs has a wide range of applications because of the unique combination of the nanoscale magnetic iron oxide core and the functional titania shell. Although some publications reported the synthesis of iron oxide-TiO_2 _core-shell nanostructure, these reported synthesis generally employed solid thick SiO_2 _interlayer. For instance, Chen et al. reported using TiO_2_-coated Fe_3_O_4 _(with a silica layer) core-shell structure NPs as affinity probes for the analysis of phosphopeptides and as a photokilling agent for pathogenic bacteria [33,34]. Recently, Wang et al. reported the synthesis of (γ-Fe_2_O_3_@SiO_2_)_*n*_@TiO_2 _functional hybrid NPs with high photocatalytic efficiency [35]. Generally, immobilization of homogeneous catalysts usually decreases the catalytic activity due to the problem of diffusion of reactants to the surface-anchored catalysts [36]. In order to increase the active surface area, hollow and ultrafine iron oxide NPs are employed in this paper. Moreover, we proposed a new utilization of magnetic NPs as a catalyst support by modifying the surface on three different-shaped amino-functionalized iron oxide NPs with an active TiO_2 _photocatalytic layer via a seed-mediate method, as shown in Figure 1. The surface amines on the magnetic iron oxide NPs can serve as functional groups for further modification of titania. We discuss the formation mechanism of iron oxide/TiO_2 _hybrid NPs. The results maybe provide some new insights into the growth mechanism of iron oxide-TiO_2 _composite NPs. It is shown that the as-synthesized iron oxide/TiO_2 _hybrid NPs display good magnetic response and photocatalytic activity. The magnetic NPs can be used as a MRCs vehicle for simply and easily recycled separation by external magnetic field application.

## Experiment

### Reagents and materials

FeCl_3_·6H_2_O, FeCl_2_·4H_2_O, FeSO_4_·7H_2_O, and KOH were purchased from Tianjin Kermel Chemical Reagent Co., Ltd. (Tianjin, China); KNO_3_, L(+)-glutamic acid (Gla, C_5_H_9_NO_4_), tetrabutyl titanate (Ti(Bu)_4_, Bu = OC_4_H_9_, CP) and methylene blue were purchased from Sinopharm Chemical Reagent CO., Ltd. (Shanghai, China); cetyltrimethylammmonium bromide (CTAB, C_19_H_42_BrN, ultrapure), MB and hexamethylenetetramine (C_6_H_12_N_4_) were purchased from Aladdin Chemical Reagent CO., Ltd. (Shanghai, China); 3-aminopropyltriethyloxysilane (APTES) were purchased from Sigma (St. Louis, MO, USA), and all the reagents are analytical pure and used as received.

### Preparation of iron oxide seeds

#### A. Spindle hematite NPs

According to Ishikava's report [37], we take a modified method to prepare the monodisperse spindle hematite NPs, in a typical synthesis, 1.8 ml of a 3.7 M FeCl_3_·6H_2_O solution was added dropwise into 4.5 × 10^-4 ^M NaH_2_PO_4 _solution at 95°C and the mixture was aged at 100°C for 12 h. The resulting precipitates were washed with a 1 M ammonia solution and doubly distilled water and finally dried under vacuum.

#### B. Hollow magnetite NPs

According to our previous report [38], in a typical synthesis, solution A was prepared by dissolving 2.02 g KNO_3 _and 0.28 g KOH in 50 mL double distilled water, solution B was prepared by dissolving 0.070 g FeSO_4_·7H_2_O in 50 mL double distilled water. Then the two solution were mixed together under magnetic stirring at a rate of ca. 400 rpm. Two minutes later, solution C (0.18 g Gla in 25 mL double distilled water) was added dropwise into the mixed solution. The reaction temperature was raised increasingly to 90°C and kept 3 h under argon (Ar) atmosphere. Meanwhile, the brown solution was observed to change black. After the mixture was cooled to room temperature, the precipitate products were magnetically separated by MSS, washed with ethanol and water two times, respectively, and then redispersed in ethanol.

#### C. Ultrafine magnetite NPs

The ultrafine magnetite NPs were prepared through the chemical co-precipitation of Fe(II) and Fe(III) chlorides (Fe^II^/Fe^III ^ratio = 0.5) with 0.5 M NaOH [39]. The black precipitate was collected on a magnet, followed by rinsing with water several times until the pH reached 6 to 7.

### Preparation of amino-functionalized iron oxide NPs

A solution of APTES was added into the above seed suspensions, stirred under Ar atmosphere at 25°C for 4 h. The prepared APTES-modified seeds were collected with a magnet, and washed with 50 mL of ethanol, followed by double distilled water for three times [40].

### Preparation of iron oxides/TiO_2 _hybrid NPs

In a typical synthesis, 0.2 g amino-functionalized seeds, 0.2 g CTAB, and 0.056 g HMTA were dissolved in 25 ml ethanol solution under ultrasonic condition at room temperature. The mixture solution was then transferred into a Teflon-lined tube reactor. Then, 1 ml Ti(Bu)_4 _dropwise added in the tube, and was kept at 150°C for 8 h.

### Photodegradation of MB

The prepared samples were weighed and added into 80 mL of methylene blue solutions (12 mg/L). The mixed solutions were illuminated under mercury lamp (OSRAM, 250 W with characteristic wavelength at 365 nm), and the MB solutions were illuminated under UV light in the photochemical reactor. The solutions were fetched at 10-min intervals by pipette for each solution and centrifuged. Then, the time-dependent absorbance changes of the transparent solution after centrifugation were measured at the wavelength between 500 and 750 nm.

### Characterization

TEM images were performed with a JEOL JEM-2010 (HT) (JEOL, Tokyo, Japan) transmission electron microscope operating at 200 kV, and the samples were dissolved in ethanol and dropped on super-thin cabon coated copper grids. SEM studies were carried out using a FEI Sirion FEG operating at 25 keV, samples were sprinkled onto the conductive substrate, respectively. Powder X-ray diffraction (XRD) patterns of the samples were recorded on a D8 Advance X-ray diffractometer (Germany) using Cu Kα radiation (*λ *= 0.1542 nm) operating at 40 kV and 40 mA and with a scan rate of 0.05° 2*θ *s^-1^. X-ray photoelectron spectroscopy (XPS) measurements were made using a VG Multilab2000X. This system uses a focused Al exciting source for excitation and a spherical section analyzer. The percentages of individual elements detection were determined from the relative composition analysis of the peak areas of the bands. Magnetic measurements were performed using a Quantum Design MPMS XL-7 SQUID magnetometer. The powder sample was filled in a diamagnetic plastic capsule, and then the packed sample was put in a diamagnetic plastic straw and impacted into a minimal volume for magnetic measurements. Background magnetic measurements were checked for the packing material. The diffuse reflectance, absorbance and transmittance spectra, and photodegradation examination of the microspheres was carried out in a PGeneral TU-1901 spectrophotometer.

## Results and discussion

### Formation mechanism and morphology

**Figure 2:**
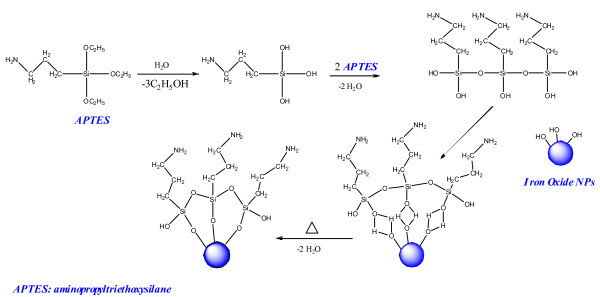
**Illustration of the functionalization process of iron oxides NPs with amino group by APTES**.

For the synthesis of the functional hybrid nanomaterials, we synthesized the colloidal solutions of iron oxides NPs with different shapes in ethanol at the first. These iron oxide NPs exhibit long sedimentation time, and are stable against agglomeration for several days. Then, iron oxides NPs were modified with amino group by APTES because silane can render highly stability and water-dispersibility, and it also forms a protective layer against mild acid and alkaline environment. As shown in Figure 2, hydroxyl groups (-OH) on the magnetite surface reacted with the -OH of the APTES molecules leading to the formation of Si-O bonds and leaving the terminal -NH_2 _groups available for immobilization of TiO_2 _[41]. The immobilization of TiO_2 _can be explained by HSAB (hard and soft acids and bases) formula [42]. As a typical hard acid, Ti ions can be combined to the terminal -NH_2 _groups (hard bases) easily, owing to there is small amount water in ethanol (95%), and then TiO_2 _will be coated on the surface of amino-functionalized iron oxide NPs by hydrolysis and poly-condensation as follows:(1)(2)

**Figure 3:**
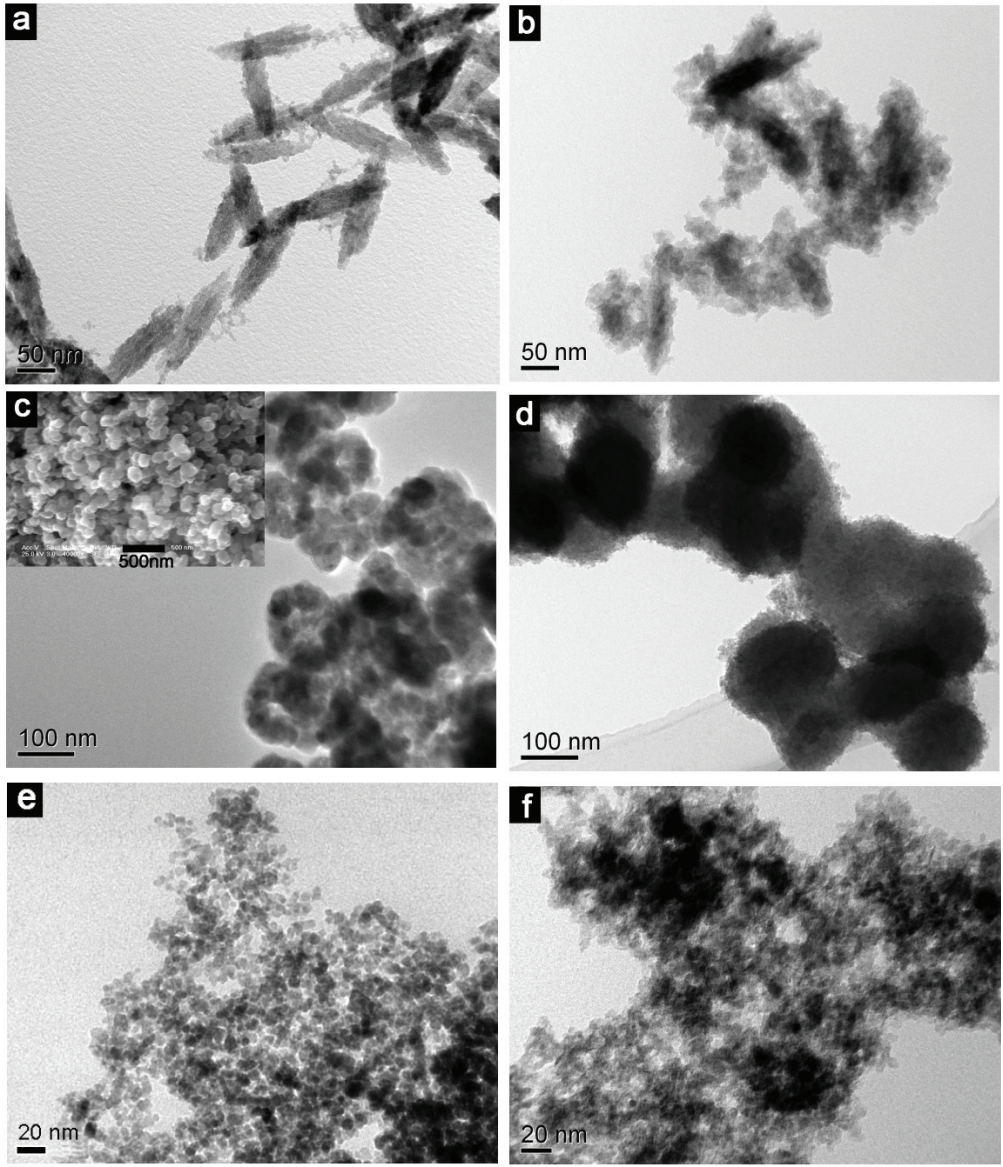
**Representative TEM images of naked iron oxides and iron oxides/TiO_2 _hybrid NPs**. The insert in **(c) **is the corresponding SEM image.

We prepared the monodisperse spindle-like iron oxide NPs by ferric hydroxide precipitate method for evaluating and verifying our experimental mechanism and functional strategies. The electron micrograph of the starting weak-magnetic spindle-like hematite NPs are shown in Figure 3a, which have longitudinal diameter in the range from 120 to 150 nm and transverse diameter (short axis) around 40 nm. After TiO_2 _coating (FT-1), the transverse diameter increased to around 50 nm, and the representative image is shown in Figure 3b. Moreover, the obvious contrast differences between the pale edges and dark centers further clearly confirms the composite structure. Therefore, the results reveal that this functional strategy for fabricating the TiO_2_-functionalized iron oxide NPs is a feasible approach. Then, two strong magnetic iron oxide NPs with different shape and diameter as seeds were employed to fabricate the magnetic TiO_2 _hybrid materials. As shown in Figure 3c, Fe_3_O_4 _NPs with an obviously hollow structure have diameters around 100 nm, and the insert field-emission SEM image illustrates the hollow NPs present sphere-like shape. In our previous report, we have confirmed that the hollow Fe_3_O_4 _NPs were formed by oriented aggregation of small Fe_3_O_4 _NPs [38]. Figure 3d shows bright field TEM image of the corresponding iron oxide NPs after the same TiO_2 _coating process (FT-2). However, the hybrid NPs present a shagginess sphere-like shape and cannot observe the hollow structure. Additionally, the diameters of hybrid NPs increased about 5 to 10 nm. The results reveal that the hollow Fe_3_O_4 _NPs have been covered by TiO_2_. Owing to the loose struture of Fe_3_O_4 _seeds, TiO_2 _will fill to its internal and surface, and finally cause the hybrid products present a solid nature. The diameter of above two different iron oxide NPs including spindle-like and hollow is relatively large, subsequently, we employ the ultrafine Fe_3_O_4 _NPs as seeds to fabricate the hybrid NPs. Figure 3e presents the TEM images of ultrafine Fe_3_O_4 _NPs without any size selection, the size is about 5 to 8 nm. By introduce the TiO_2_, the as-obtained products (FT-3) exhibit an aggregated nature and the ultrafine Fe_3_O_4 _NPs dispersing in the TiO_2 _matrix, as shown in Figure 3f.

### Structure and composition

**Figure 4:**
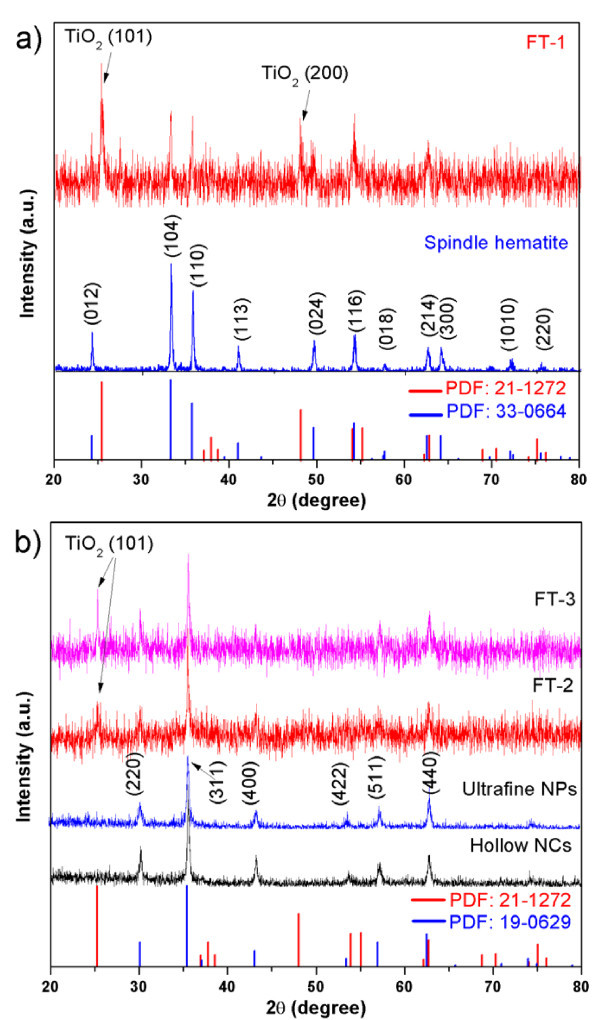
**XRD patterns**. Patterns of the as-prepared spindle-like α-Fe_2_O_3 _NPs and FT-1 **(a)**, as-prepared hollow and ultrafine Fe_3_O_4 _NPs, FT-2 and FT-3 **(b)**.

XRD and XPS surface analysis was used to further confirm the structure and composition of iron oxides/TiO_2 _hybrid NPs. Figure 4a shows the XRD patterns of the as-synthesized α-Fe_2_O_3 _seeds and α-Fe_2_O_3_/TiO_2 _(FT-1). From the XRD patterns of α-Fe_2_O_3 _seeds, it can be seen that the diffraction peaks conformity with that of rhombohedral α-Fe_2_O_3 _(JCPDS no. 33-0664, show in the bottom). After coating, compared with that data of JCPDS no. 33-0664 and JCPDS no. 21-1272 (pure anatase TiO_2 _phase), the (101) and (200) peaks of anatase TiO_2 _can be found in FT-1, suggesting that α-Fe_2_O_3_/TiO_2 _composite NPs are successfully fabricated by this method. Figure 4b shows the XRD patterns of the as-synthesized Fe_3_O_4 _seeds and Fe_3_O_4_/TiO_2 _(FT-2 and FT-3). All peaks in the XRD patterns of both seeds can be perfectly indexed to the cubic Fe_3_O_4 _structure (JCPDS no. 19-0629, show in the bottom). After coating, the (101) peak of anatase TiO_2 _can be clearly found in FT-2 and FT-3, suggesting that Fe_3_O_4_/TiO_2 _hybrid NPs are successfully synthesized.

**Figure 5:**
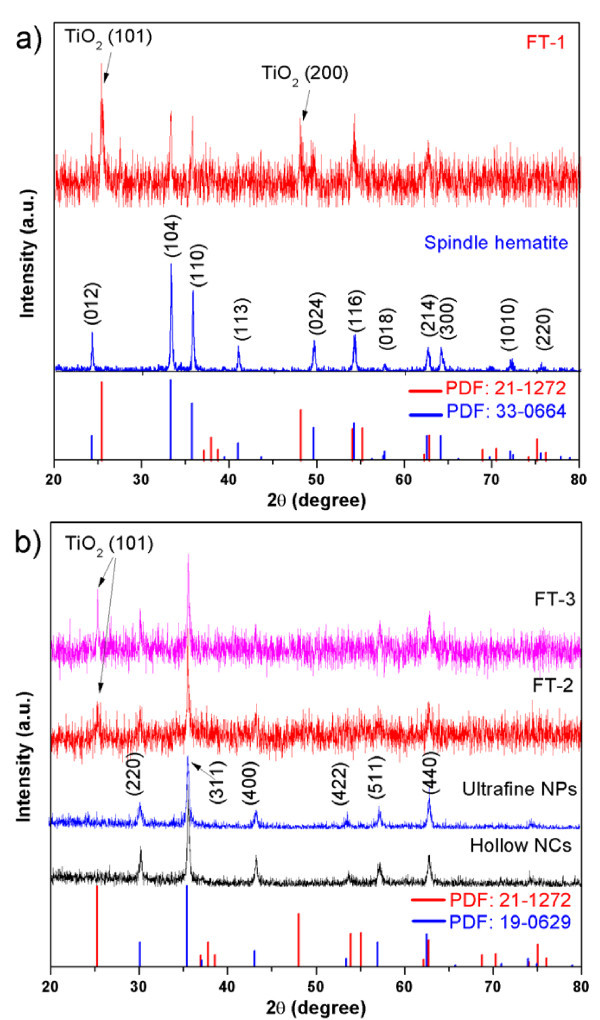
**XPS spectra of the naked, amino-functionalized, and titania coating ultrafine Fe_3_O_4 _NPs**. XPS spectra for ultrafine Fe_3_O_4 _NPs (curve a), APTES-coated ultrafine Fe_3_O_4 _NPs (curve b) and ultrafine Fe_3_O_4_/TiO_2 _hybrid NPs (curve c) comparison **(a)**, the regions for Fe 2*p ***(b)**, Si 2*p ***(c)**, O 1*s ***(d)**, and C 1*s ***(e)**, comparison respectively.

Figure 5 is the typical XPS spectra of the naked, amino-functionalized, and titania coating ultrafine Fe_3_O_4 _NPs, where part (a) is the survey spectrum and parts (b) to (d) are the high-resolution binding energy spectrum for Fe, Si, O, and Ti species, respectively. According to the survey spectrum, the elements of Fe, O, and C are found in the naked ultrafine Fe_3_O_4 _NPs, of which the element of C is found on the surface as the internal reference, and the elements of Fe and O arise from the components of Fe_3_O_4_. The new signals of N 1*s*, Si 2*s*, and Si 2*p *are observed in APTES-coated Fe_3_O_4 _NPs, and the new signal of Ti 2*p *signals is observed in FT-3 hybrid NPs. These results indicate that the FT-3 are composed of two components, silane functionalized Fe_3_O_4 _and TiO_2_. It is noteworthy that many studies demonstrated that if particles possessed a real core and shell structure, the core would be screened by the shell and the compositions in the shell layer became gradually more dominant, the intensity ratio of the shell/core spectra would gradually increase [43-47]. The gradually subdued XPS signals of Fe after TiO_2 _coating are discerned in Figure 5b. APTES coating increases the intensity of carbon and oxygen, and decreases the concentration of Fe; further TiO_2 _coating decreases the intensity of silicon and Fe (as shown in Figure 5b, c). Therefore, after TiO_2 _coating, corresponding XPS signals of Fe, and Si rule also are decreased, C and O do not match with this rule due to the formation of TiO_2 _and surfactant impurities (as shown in Figure 5d, e). Additionally, interactions should exist among APTES-coated Fe_3_O_4 _NPs and titania which cause the shift of binding energy of Fe. Usually, XPS measures the elemental composition of the substance surface up to 1 to 10 nm depth. Therefore, XPS could be regarded as a bulk technique due to the ultrafine particles size of the FT-3 (less than 10 nm). The XPS result indicates that the amino-functionalized Fe_3_O_4 _seeds have been coated by a TiO_2 _layer, thus greatly reducing the intensity signals of the element inside. Table 1 lists the binding energy values of Fe, Si, O, N, and Ti resolved from XPS spectra of the above three different NPs. In three cases, the value of binding energy of Fe 2*p *and other elements are very close to the standard binding energy values. Relative to the standard values [48], the binding energy values in FT-3 have decreased and this result is in agreement with the previous discussions.

**Table 1:** Standard binding energy values

**Samples**^**a**^	**Fe 2p**_**3/2**_	O 1*s*	Si 2*p*	N 1*s*	**Ti 2*p***_**3/2**_
Naked Fe_3_O_4 _nanoparticles	710.9	531.5			
APTES-coated Fe_3_O_4 _nanoparticles	710.5	531.5	102.5	399.5	
Hybrid nanoparticles (FT-3)	710.0	530.0	101.4	400.7	458.3
Standard value	710.5^b^	531.4^c^, 529.9^d^	103.3^e^	399.8^f^	458.8^g^

Standard binding energy values for Fe 2*p*, Si 2*p*, N 1*s*, O 1*s*, and Ti 2*p *and those resolved in the naked, amino-functionalized, and titania coating ultrafine Fe_3_O_4 _nanoparticles. ^a^Unit for binding energy: eV; ^b^Fe in Fe_3_O_4_; ^c^O in Fe_3_O_4_; ^d^O in TiO_2_; ^e^Si in SiO_2_; ^f^N in N-C group; ^g ^Ti in TiO_2_, *Δ *= 5.54 eV

**Figure 6:**
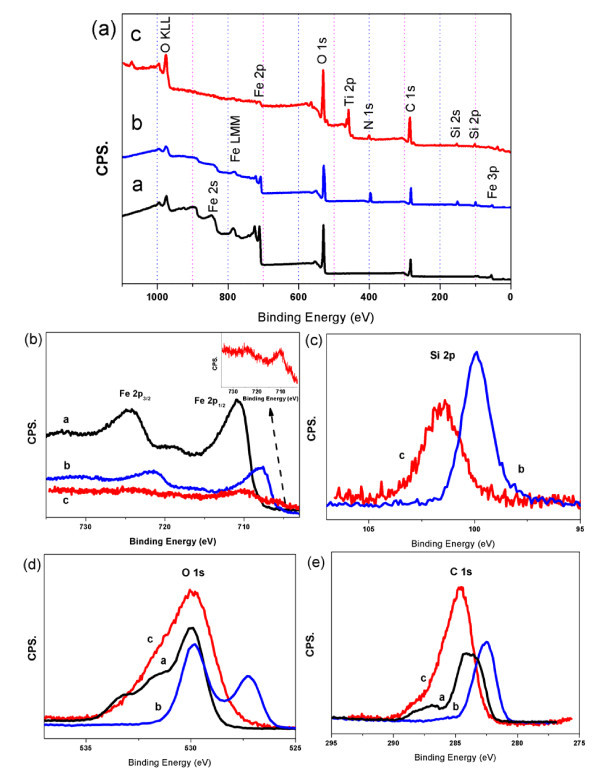
**XPS spectra of the FT-1, FT-2, and FT-3**. XPS spectra for FT-1 (curve a), FT-2 (curve b), and FT-3 (curve c) comparison **(a)**, the regions for C 1*s ***(b)**, O 1*s ***(c)**, N 1*s ***(d)**, Si 2*p ***(e)**, Fe 2*p ***(f)**, and Ti 2*p ***(g)**, comparison respectively.

Furthermore, XPS surface analysis is also used to quantify the amount of titanium and iron present in the near surface region of the three different hybrid NPs. Figure 6 is the typical XPS spectra of the FT-1, FT-2, and FT-3, where part (a) is the survey spectrum and parts (b)-(d) are the high-resolution binding energy spectrum for Fe, Si, O, C, N, and Ti species, respectively. According to the survey spectrum, all hybrid NPs exhibited typical binding energies at the characteristic peaks of Ti 2*p*, Fe 2*p*, Si 2*p*, N 1*s *and O1*s *in the region of 458, 710, 103, 400, and 530 eV, respectively. Details of the XPS surface elemental composition results of as-obtained products are shown in Table 2. The XPS data of the titanium-to-iron ratio of hybrid NPs is calculated in which the elemental composition ratio of FT-1, FT-2, and FT-3 (titanium/iron) are about 2:1, 3.5:1, and 5.5:1. The results reveal that the quantity of Ti element is higher than that of Fe element on the surface of samples. That is, it may deduce that iron oxide NPs have been coated by TiO_2_. In all hybrid NPs, the amount of oxygen to titanium or iron calculated from XPS data is about 5:1, this results is in agreement with the other reports [49]. Nevertheless, the combined results from TEM and XPS suggest that the synthesized hybrid NPs are composed of amino-functionalized iron oxide NPs and TiO_2_.

**Table 2:** Surface elemental composition and XPS binding energies of FT-1, FT-2, and FT-3

	Chemical composition (%); in parentheses, binding energy (eV)					Atomic ratio	
Samples	Ti 2*p*	Fe 2*p*	O 1*s*	N 1*s*	Si 2*p*	Ti/Fe	O/FeTi
FT-1	2.87 (457.7)	1.38 (709.2)	29.08 (529.4)	3.96 (400.3)	3.80 (101.5)	2.08	6.84
FT-2	4.72 (457.8)	1.25 (709.3)	27.13 (529.5)	4.10 (401.2)	2.60 (101.0)	3.78	4.54
FT-3	5.75 (458.3)	1.02 (710.0)	33.53 (530.0)	4.38 (400.7)	5.48 (101.4)	5.63	4.95

### Magnetic and magnetic response properties

**Figure 7:**
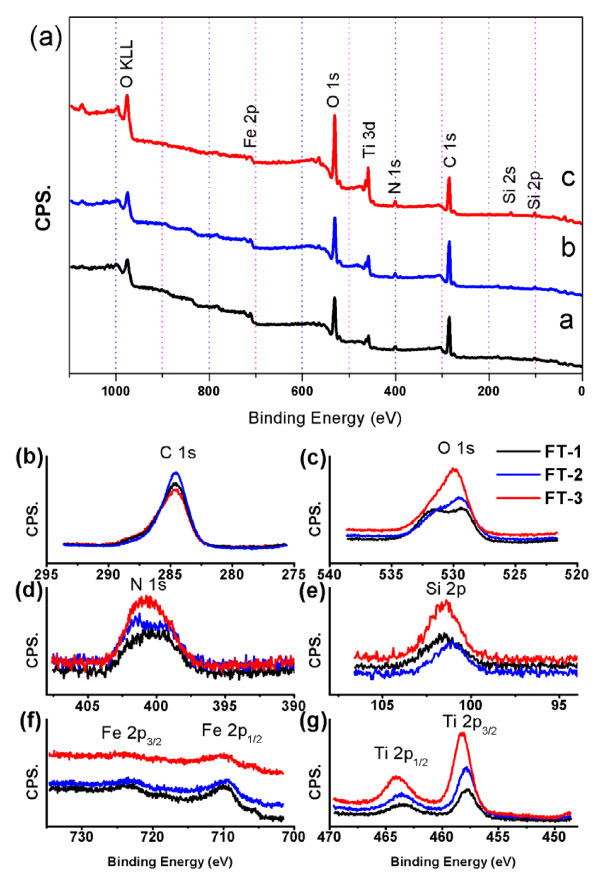
**Magnetization vs. filed dependence curves of iron oxides and hybrid NPs**. Recorded at T = 300 K. Insert shows the M-H curve of FT-1 samples.

Magnetic measurements of the hybrid NPs were performed on a SQUID magnetometer. As shown in Figure 7, hysteresis loops demonstrate that FT-2 and FT-3 have no hysteresis, the forward and backward magnetization curves overlap completely and are almost negligible. Moreover, the NPs have zero magnetization at zero applied field, indicating that they are superparamagnetic at room temperature, no remnant magnetism was observed when the magnetic field was removed [50]. Superparamagnetism occurs when the size of the crystals is smaller than the ferromagnetic domain (the size of iron oxide NPs should less than 30 nm), the size of the ultrafine Fe_3_O_4 _component in our product is less than 10 nm, and the hollow Fe_3_O_4 _is consist of small magnetite NPs, there are reasonable to suppose that the hybrid NPs showed superparamagnetic behavior. The results reveal that the products have been inherit the superparamagnetic property from the Fe_3_O_4 _NPs, and the saturation magnetization value (*M*_s_) of naked hollow Fe_3_O_4 _and ultrafine Fe_3_O_4 _is 89.2 and 72.1 emu/g, respectively. After TiO_2 _coating, the corresponding value of *M*_s _decreases to 16.2 and 5.0 emu/g, respectively. The *M*_s _decreased significantly after coating with TiO_2 _due to the surface effect arising from the non-collinearity of magnetic moments, which may be due to the coated TiO_2 _is impregnated at the interface of iron oxide matrix and pinning of the surface spins [51]. Moreover, this decrease in magnetic behavior is very close to other reports [52,53]. As the most stable iron oxide NPs in the ambient conditions, the magnetic properties of hematite are not well understood [54-56]. We checked the magnetic properties of FT-1 hybrid NPs, the *M*_s _is about 2 × 10^-4 ^emu/g, and the composite NPs exhibit a typical ferromagnetism. Thereby, as a weak magnetic hybrid NPs, FT-1 cannot be separate by common magnet.

**Figure 8:**
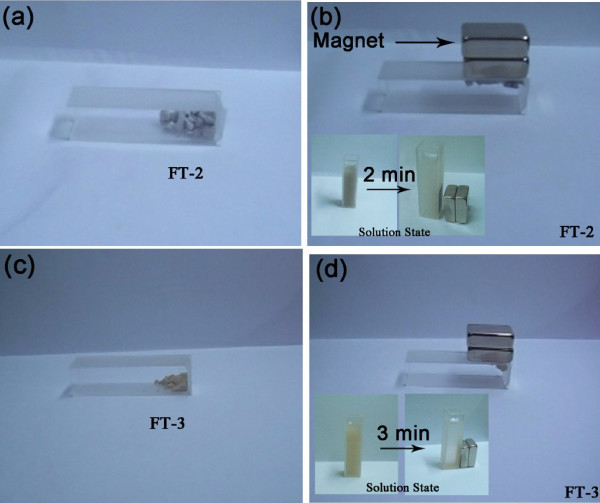
**Photographs showing the magnetic separation of the FT-2 and FT-3 in solid and solution state**. At the presence of magnet (take from the MSS).

We checked the magnetic responsibility of FT-2 and FT-3 hybrid NPs under the external applied magnetic field by a common magnet. As shown in Figure 8, both hybrid NPs gather quickly without residues left in the solid and solution state when the magnet presence. The gathered hybrid NPs can be redispersed in the solution easily by a slight shake. The results illustrate that the hybrid NPs display a good magnetic response, and this is also important for the industrial application in water cleaning as MRCs for preventing loss of materials and save cost.

### Optical adsorption and photocatalytic properties

**Figure 9:**
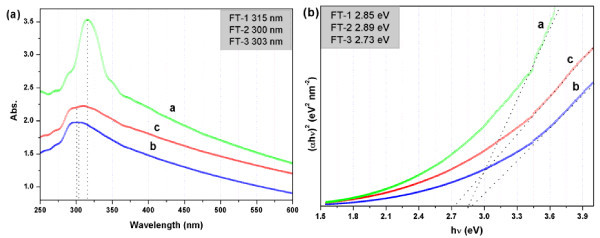
**UV-vis absorbance spectrum and bandgap energy**. UV-vis absorbance spectrum **(a) **and bandgap energy **(b) **of FT-1 (curve a), FT-2 (curve b) and FT-3 (curve c) hybrid NPs.

The three different hybrid NPs were further characterized by UV-vis absorption spectra to compare their optical adsorption properties and the results are shown in Figure 9a. The spectra highlight a strong adsorption in the UV region, the results are in agreement with the other reports [57,58]. It is noteworthy that the hybrid NPs with different morphology (at same concentration) will cause the difference of adsorption intensity and peak location. Due to the small dimensions of semiconductor NPs, a discretization of the bandgap occurs with decreasing particle size, leading to smaller excitation frequencies. A blue shift of FT-3 is observed in the extinction behavior, and the absorption edge is positioned at smaller wavelengths [59]. The result confirms that the diameter of FT-1 hybrid NPs is large than the other two different types hybrid NPs. Additionally, a concomitant tail can be clearly observed in the visible region of the absorption curve owing to scattering losses induced by the large number of inorganic NPs in the composite nanostructure [60].

In order to calculate the bandgap of hybrid NPs, the relationship between the absorption coefficient (*α*) and the photon energy (hν) have been given by equation as follows: *α*hv = *A*(hv-*E*_*E*_)^*m*^, where *A *is a constant, *E*_g _is the bandgap energy, hν is the incident photon energy and the exponent m depends on the nature of optical transition. The value of *m *is 1/2 for direct allowed, 2 for indirect allowed, 3/2 for direct forbidden, and 3 for indirect forbidden transitions [61]. The main mechanism of light absorption in pure semiconductors is direct interband electron transitions. The absorption coefficient α has been calculated from the Lamberts formula [62], $\alpha =\frac{1}{t}\ln\left(\frac{1}{T}\right)$, where *T *and *t *are the transmittance (can be directly measured by UV-vis spectra) and path length of the colloids solution (same concentration), respectively. A typical plot of (*α*hν)^2 ^versus photon energy (hν) for the samples are shown in Figure 9b. The value of FT-1, FT-2, and FT-3 is 2.85, 2.89, and 2.73 eV, respectively.TiO_2 _is important for its application in energy transport, storage, and for the environmental cleanup due to its well known photocatalytic effect with a bandgap of 3.2 eV [63]. Comparing with the pure TiO_2 _NPs, the bandgap of hybrid NPs is obviously decreased, and the absorption edge generates obvious red shift. This red shift is attributed to the charge-transfer transition between the electrons of the iron oxide NPs and the conduction band (or valence band) of TiO_2 _[64]. Iron oxide NPs can increase energy spacing of the conduction band in TiO_2 _and finally lead to the quantization of energy levels and causes the absorption in the visible region. The other is that amino groups can act as a substitutional dopant for the place of titanium and change metal coordination of TiO_2 _and the electronic environment around them [65]. Similar phenomenon of red shift in the bandgap for iron oxide/TiO_2 _hybrid NPs were also found by other reports [53,65-67].

**Figure 10:**
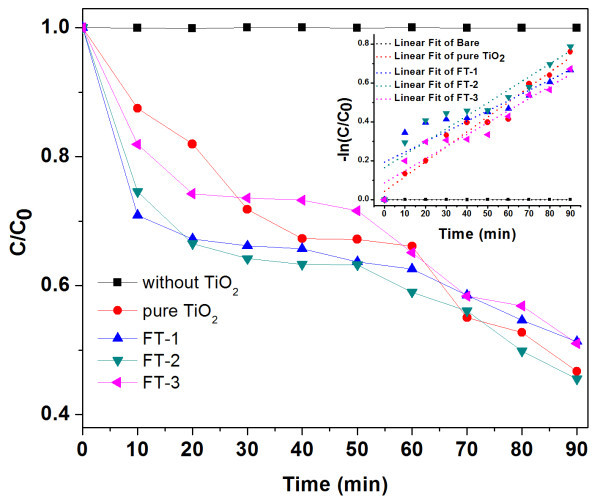
**Changes of MB concentration photocatalytic degradation in the presence of samples**. **(a) **Without samples, **(b) **pure TiO_2 _(5 nm), **(c) **FT-1, **(d) **FT-2, and **(e) **FT-3, and the insert is the correspondingly logarithmic coordinate versus time and liner fitting results.

The photocatalytic activity was examined by a colorant decomposition test using MB, which is very stable chemical dye under normal conditions. In general, absorption spectra can be used to measure the concentration changes of MB in extremely dilute aqueous solution. The MB displays an absorption peak at the wavelength of about 664 nm. Time-dependent photodegradation of MB is shown in Figure 10. It is illustrated that MB decomposes in the presence of magnetic TiO_2 _hybrid materials. Generally, the pure TiO_2 _NPs can decompose 40% MB in 90 min [68-70]. In our previous report, the pure TiO_2 _NPs with a average diameter of 5 nm can be decomposed 53% MB in 90 min [71]. However, in our system, 49.0%, 56.5%, and 49.6% MB decomposed by FT-1, FT-2, and FT-3 in 90 min, respectively. The result reveals that the introduction of iron oxide NPs not only improve the photocatalytic activity but also employ the corresponding magnetic properties from itself. Thus, the as-synthesized magnetic hybrid NPs with high photocatalytic efficiency are very potentially useful for cleaning polluted water with the help of magnetic separation. The photocatalytic degradation generally follows a Langmuir-Hinshelwood mechanism, which could be simplified as a pseudo-first order reaction as follows [72,73]: $r=-\frac{dC_{t}}{dt}=kCt$, where *r *is the degradation rate of reactant, *C *is the concentration of reactant, *k *is the apparent reaction rate constant. The *k *for FT-1, FT-2, and FT-3 was 1.066% min^-1^, 1.331% min^-1^, 1.054% min^-1^, respectively. It was surprising that the FT-2 exhibited such higher activity. This may be explained by light absorption capability of the FT-2 due to their rough shell contributes to the good photocatalytic activity. Compared to smooth surface, the rough surface layers can absorb more light because the UV-vis light can have multiple-reflections among the shagginess surface structure [74].

## Conclusions

In summary, MRCs have been fabricated via a facile seed-mediate technology. These iron oxide/TiO_2 _hybrid NPs were synthesized in a stepwise process. First, three different shapes of naked iron oxide NPs were prepared. Next, amino groups encapsulated iron oxide NPs are synthesized by APTES modification. Finally, the iron oxide/TiO_2 _hybrid NPs can be obtained after the TiO_2 _coating. The FT-2 and FT-3 hybrid NPs show superparamagnetic and both display good photocatalytic properties. This MRCs combination of the photocatalysis properties of TiO_2 _and the superparamagnetic property of Fe_3_O_4 _NPs endows this material with a bright perspective in purification of polluted wastewater. Additionally, this work also discusses the formation mechanism and potentially provided a general method for synthesizing nanocomposites of magnetic iron oxide NPs and other functional NPs, which may find wider applications besides in photocatalysis.

## Competing interests

The authors declare that they have no competing interests.

## Authors' contributions

WW participated in the materials preparation, data analysis and drafted the manuscript. SZ, XX and RF participated in the sample characterization. CZ participated in its design and coordination. All authors read and approved the final manuscript.
